# Cell cycle-dependent resolution of DNA double-strand breaks

**DOI:** 10.18632/oncotarget.6644

**Published:** 2015-12-17

**Authors:** Susanna Ambrosio, Giacomo Di Palo, Giuliana Napolitano, Stefano Amente, Gaetano Ivan Dellino, Mario Faretta, Pier Giuseppe Pelicci, Luigi Lania, Barbara Majello

**Affiliations:** ^1^ Department of Biology, University of Naples ‘Federico II’, Naples, Italy; ^2^ Department of Molecular Medicine and Medical Biotechnologies, University of Naples ‘Federico II’, Naples, Italy; ^3^ Department of Experimental Oncology, European Institute of Oncology, Milan, Italy; ^4^ Department of Oncology and Haemato-oncology, University of Milan, Italy

**Keywords:** cell-cycle, DSB repair, site-specific DSBs, AsiSI restriction enzyme

## Abstract

DNA double strand breaks (DSBs) elicit prompt activation of DNA damage response (DDR), which arrests cell-cycle either in G_1_/S or G_2_/M in order to avoid entering S and M phase with damaged DNAs. Since mammalian tissues contain both proliferating and quiescent cells, there might be fundamental difference in DDR between proliferating and quiescent cells (or G_0_-arrested). To investigate these differences, we studied recruitment of DSB repair factors and resolution of DNA lesions induced at site-specific DSBs in asynchronously proliferating, G_0_-, or G_1_-arrested cells. Strikingly, DSBs occurring in G_0_ quiescent cells are not repaired and maintain a sustained activation of the p53-pathway. Conversely, re-entry into cell cycle of damaged G_0_-arrested cells, occurs with a delayed clearance of DNA repair factors initially recruited to DSBs, indicating an inefficient repair when compared to DSBs induced in asynchronously proliferating or G_1_-synchronized cells. Moreover, we found that initial recognition of DSBs and assembly of DSB factors is largely similar in asynchronously proliferating, G_0_-, or G_1_-synchronized cells. Our study thereby demonstrates that repair and resolution of DSBs is strongly dependent on the cell-cycle state.

## INTRODUCTION

Eukaryotic genome is constantly being challenged by various endogenous and exogenous insults. These damaging events include crosslinks, base modifications, base mismatches, stalled replication forks, single-strand breaks (SSBs), and particularly dangerous double-strand breaks (DSBs). To deal with such dangerous insults, eukaryotes possess an array of distinct pathways to monitor and repair damaged DNA.

The initial phases of DSB recognition and recruitment of repair factors are now quite elucidated. Following DSB the MRE11/RAD50/NBS1 complex senses a DSB within seconds and then activates PI3K-like kinases ATM (ataxia-telangiectasia mutated) protein kinase, a large Ser/Thr kinase of the PI3K-like kinase family, which also includes DNA-PKcs (DNA-dependent protein kinase catalytic subunit), and ATR [1–4]. ATM then phosphorylates histone H2AX on Ser139 (named γH2AX when phosphorylated) in DSB adjacent chromatin. The primary function of γH2AX is to recruit its decoder, MDC1 (mediator of DNA damage checkpoint protein 1), which recognizes the phosphorylated Ser139 epitope on γH2AX. γH2AX-bound MDC1 recruits in turn more MRN complexes (via an interaction with NBS1) and thus initiates a positive ATM feedback loop that leads to the amplification of the γH2A.X chromatin domain [5–8]. Concomitant with the assembly of DNA repair factors at DNA lesion, DSB response activates DNA-damage checkpoints (DDR), and diffusible signaling events that can arrest cell cycle progression either in G_1_ or G_2_ to allow for DNA repair and prevent transmission of damaged DNA to daughter cells (9,10). However, it must be emphasized that the tissue and organs of mammals consist of different cell types, including dividing, non-dividing and stem cells that coexist in several tissues, in separate yet adjoining locations [11]. Normal mammalian cells possess unique regulatory mechanisms to shift from a quiescent state to a proliferative state and dysregulation of these mechanisms might result in malignant transformation. Cellular quiescence and the capacity to enter the proliferation cycle are critical for maintaining tissue homeostasis [12, 13].

During interphase, DSB can be repaired in a cell-cycle dependent manner by two major mechanisms: classical non-homologous end joining (NHEJ, during G_1_ phase) or homologous recombination repair (HR, mainly in S-G_2_ phases) [14–16]. Several studies unveiled cell cycle-regulated circuits that govern DSB repair pathway choice to ensure that NHEJ dominates in G_1_ and HR is favored from S phase onward [17–21]. Although cell-cycle phase contributes to this choice, these pathways coexist in S- and G_2_-phases, thus implying that other factors participate in this decision such as chromatin context in which DSB occurs [22–24].

It is now well established that DDR differs in mitotic and interphase cells [25, 26]. It has been shown that DDR is dampened during mitosis. Cells have evolved mechanisms to suppress DSB repair during M phase to prevent genome instability [27, 28]. Clearly, different molecular mechanisms involved in DNA repair occurring at specific cell cycle phases have been evolved, and recruitment of DSB repair factors and resolution of DNA lesions induced at site-specific DSBs occurring during different phases of the cell cycle could be instrumental to investigate these differences.

To avoid potential anomalies associated with transformed cell lines, we produced a cellular system suitable to the induction of specific DSBs in the immortalized non-tumorigenic epithelial cell line MCF10A. We stable transfected these cells with a well-documented AsISI-inducible vector that express the 8-base restriction endonuclease AsiSI fused to a modified oestrogen receptor ligand binding domain that induces nuclear localization of the enzyme after administration with 4hydroxytamoxifen (4OHT) causing the rapid and reproducible induction of about 150 sequence-specific DSBs across the genome [23, 29–31]. This system (MCF10A-AsiSI-ER) offers the opportunity to study the wave of repair events occurring at defined stages of the cell cycle in a defined and reproducible manner.

We found the DSBs occurring in G_0_ quiescent cells are irreparable with a sustained activation of the p53-pathway. Conversely, re-entry into cell cycle of damaged G_0_-arrested cells shows a delayed clearance of recruited DNA repair factors bound at DSBs, indicating inefficient repair. This study thereby demonstrates the crucial role of cell cycle phases in repair and resolution of DSBs.

## RESULTS

### Induction of specific DSBs in non-tumorigenic epithelial MCF10A cells

**Figure 1:**
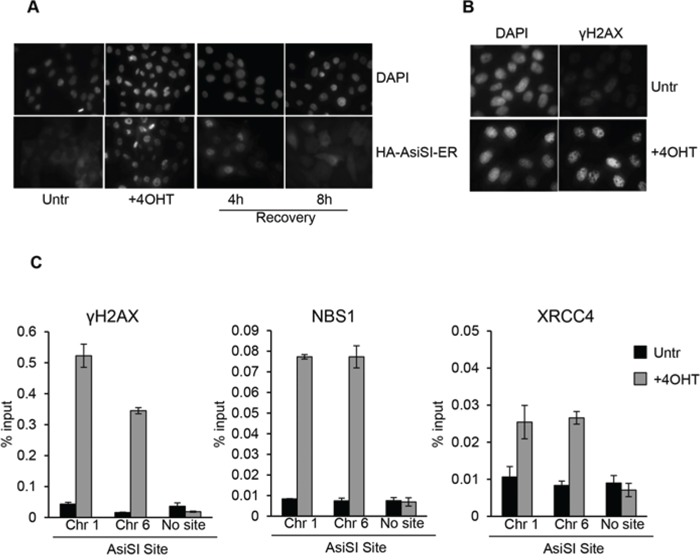
4OHT treatment triggers DSBs formation at AsiSI sites in MCF10A **A.** MCF10A-AsiSI-ER cells were treated for 2 h with 4OHT or vehicle (Untr) and then released into fresh medium for 4 and 8 h (Recovery). Cells were fixed and processed for anti-HA immunofluorescence and DAPI staining. **B.** MCF10A-AsiSI-ER cells were treated for 2 h with 4OHT and stained with anti-γH2AX antibody. DAPI staining of nuclei is shown. **C.** MCF10A-AsiSI-ER cells were treated as above and ChIP experiments were performed using antibodies against γH2AX, NBS1 and XRCC4. Real-time qPCR was done on ChIP materials using primers listed in Supplementary Table 2. Amplicon far from any AsiSI site was analyzed as negative control. Data are from independent experiments with SD (*n* = 3).

To investigate DSB damage and avoid potential anomalies associated with transformed cell lines, we sought to produce a cellular system suitable to the induction of specific DSBs in the immortalized non-tumorigenic epithelial cell line MCF10A. To this end MCF10A cells were transduced with a retroviral vector expressing the fusion protein between the HA-tagged AsiSI restriction enzyme and a modified hormone-binding domain from the estrogen receptor. Following drug selection, one cell clone was isolated (named MCF10-AsiSIER) and the effects of 4OHT administration at different time points were analyzed by indirect immunofluorescence. Exposure to 4OHT for 2 hours resulted in nuclear accumulation of the AsiSI fusion protein, as detected by anti-HA-tag antibodies (Figure 1A). This was accompanied by a significant increase in the number of DNA damaged foci, visualized with antibodies against S139-phosphorylated histone γH2AX (Figure 1B). 4OHT was removed from the medium after 2 hours and cells cultivated for additional 4 and 8 hours (Recovery). As shown in Figure 1A, the nuclear localization of HA-AsiSI-ER was strongly reduced after 4 hours of recovery, and barely detectable after 8 hours, indicating that the HA-AsiSI-ER endonuclease was not active anymore (Figure 1A). The generation of the MCF10-AsiER clone enabled us to investigate recruitment of DNA damaging factors at specific DSBs by using ChIP-based approaches. As initial test we focused on the AsiSI sites on chromosomes 1 and 6 at which γH2AX recruitment had been observed and documented by treatment of U2OS AsiSI cells [29]. We conducted ChIP assays with antibodies against some of the DDR components and we used sequences of published primer sets (listed in Table S2). As illustrated in Figure 1C, we observed increased enrichment of γH2AX, NBS1, and XRCC4 at the Chr1 and Chr6 AsiSI sites. These results confirm that recruitment of these factors in MCF10-AsiER paralleled the effects observed in previously described clonal population of U2OS-AsiER cells.

### DSBs induce DDR activation followed by efficient repair in MCF10A proliferating cells

**Figure 2:**
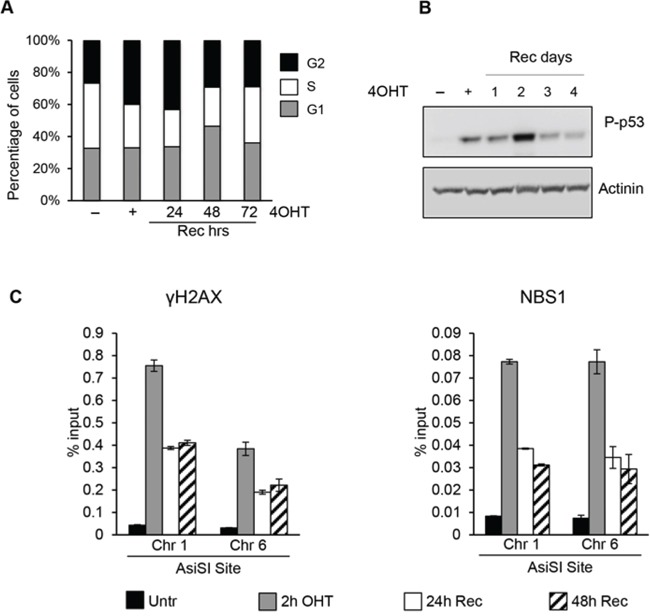
AsiSI-induced DSBs trigger DDR activation followed by efficient wave of repair **A.** Cell cycle distribution of asynchronously growing MCF10A-AsiSI-ER treated for 2h with 4OHT then released into fresh medium and collected as indicated. DNA content of propidium iodide stained cells was determined by flow cytofluorimetry. **B.** Total cell extracts from proliferating MCF10A-AsiSI before and at the indicated times after 4OHT removal were probed with anti-phospho-p53 and normalized for actinin. **C.** ChIP against γH2AX and NBS1 in MCF10A-AsiSI-ER treated for 2h with 4OHT then released into fresh medium, collected as indicated and analyzed by qPCR. Data are from independent experiments with SD (*n* = 3).

Following the generation of DSBs, DDR promotes cellular DNA-repair activities with a concomitant transient arrest of cell-cycle progression (checkpoint function) until DNA damage has been removed. To analyze the transient arrest of cell-cycle progression following induction of DSBs, proliferating MCF10-AsiSIER cells were treated for 2 hours with 4OHT and then allowed to recover in the absence of 4OHT for 24, 48 and 72 hours. Samples were analyzed for cell-cycle distribution, DDR activation, and ChIP accumulation of γH2AX and NBS1 at specific AsiSI sites. Cell cycle analysis showed that AsiSI-dependent DSBs induced a significant G2 arrest, which was completely resolved after 72hr of Recovery (Figure 2A). As shown in Figure 2B, p53-Ser15 phosphorylation increased after 4OHT treatment and its levels decreased 3 days after the removal of the DNA damage insult.

DDR cascade begins with the detection of DSBs by the MRN (MRE11-RAD50-NBS1) complex, which recruits and activates different PIKK kinases (ATM, ATR and DNA-PK), each capable to phosphorylate H2AX at Ser139 [3–5]. To analyze the efficiency of these steps detecting DSBs and to monitor the resolution of DNA damage-associated γH2AX and NBS1 accumulation at defined AsiSI sites we performed ChIP with anti-γH2AX and -NBS1 antibodies. Following the robust increase of γH2AX and NBS1 signals at the AsiSI sites after 4OHT treatment, we observed their progressive reduction within 24 hours (Figure 2C and Supplementary Figure 1).

Collectively, these data indicate that induction of DSBs in asynchronously proliferating MCF10 cells promotes a robust DDR activation, which is followed by an efficient wave of repair leading to a progressive reduction of DDR after DSBs onset.

### DSBs in quiescent MFC10 cells are irreparable and cause a sustained activation of the p53-pathway

**Figure 3:**
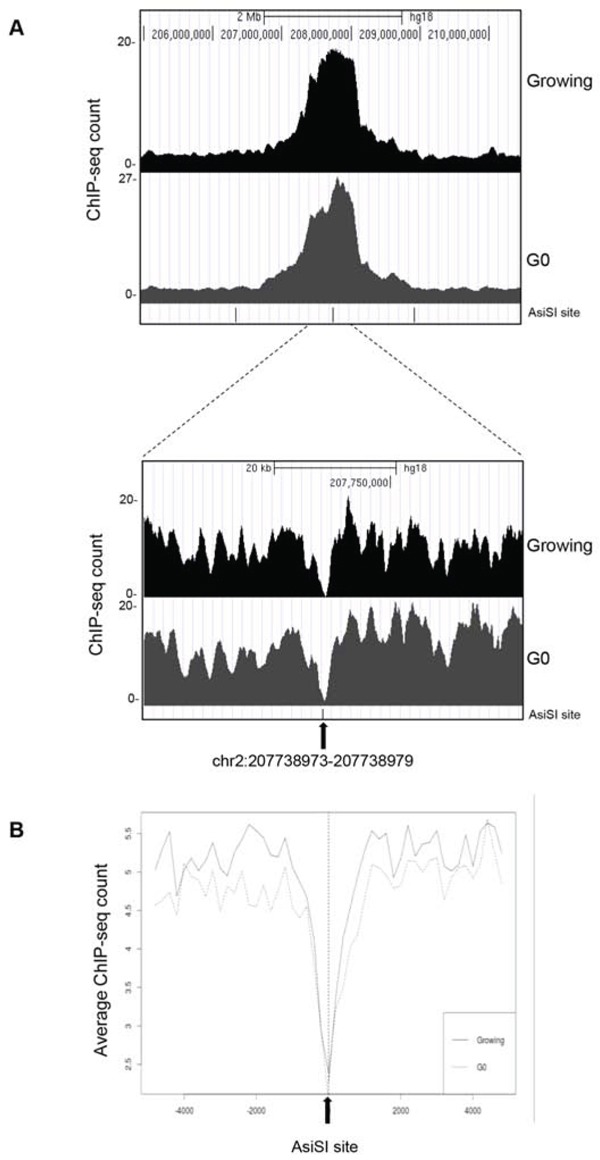
ChIP-seq analyses in proliferating and G_0_-arrested MCF10A-AsiSI-ER cells after 4OHT treatment (2 h), using anti-γH2AX antibody Panel **A.** show the profiles of γH2AX around a selected AsiSI site in both proliferating and G_0_-arrested cells. **B.** Averaged γH2AX signals of proliferating and G_0_ cells over a 10-kb windows and centered at the AsiSI site.

**Figure 4:**
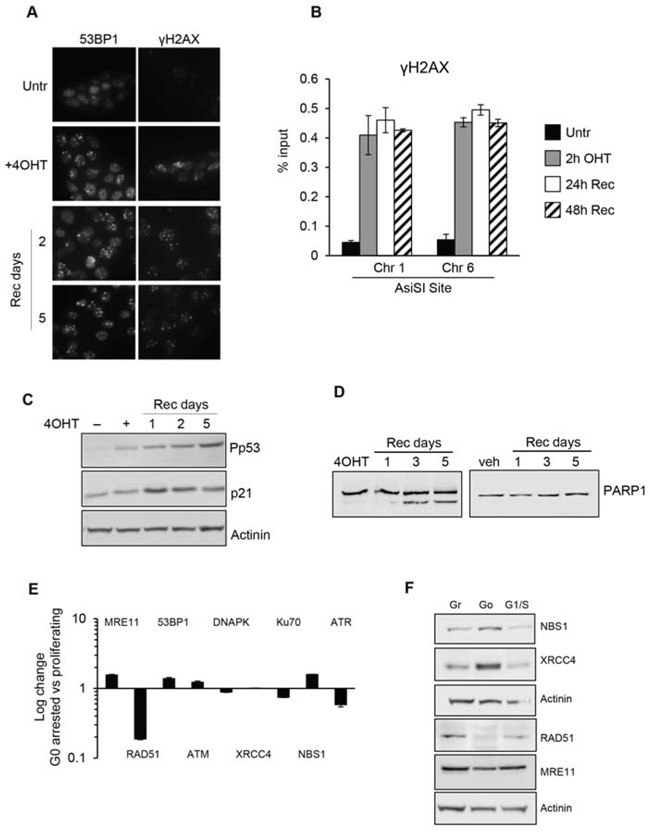
G_0_-arrested MCF10-AsiSIER cells lack DNA repair proficiency **A.** MCF10A-AsiSIER cells were arrested in G_0_ phase through grow factors deprivation for 40h, treated with 4OHT for 2h then kept in medium without grow factors, and analyzed at the indicated times after 4OHT removal by immunofluorescence with anti-53BP1 and anti- γH2AX antibodies, respectively. **B.** Recruitment of γH2AX at AsiSI sites (Chr. 1 and 6) was determined by ChIP assays. **C.** Western blotting was performed using phospho-p53 antibodies and p21. **D.** PARP1 detection of both full-length and cleaved protein fragments; western blotting of G_0_-arrested MCF10-AsiSI-ER treated with 4OHT or vehicle, collected at the indicated time points after 4OHT removal. **E.** DDR factors mRNAs expression analysis of G_0_-arrested MCF10-AsiSI-ER through quantitative RT-PCR. Expression profiles were normalized against proliferating cells. **F.** Western blot of protein extracts of Growing, G_0_ and G1/S MCF10-AsiSI-ER cells using the indicated antibodies. Actinin has been probed as loading control for different blots.

In mammalian tissues, cells are in both proliferating and quiescent states depending on the given tissue and these two different populations may also coexist in several tissues, in separate yet adjoining locations. However, comparative study of these two distinct cell cycle states regarding the capability to sense and resolve DNA DSB damaging insults has been poorly characterized. To address this issue and investigate if quiescent or proliferating cells equally sense and resolve DSBs over time, we took advantage of the MCF10AsIER cells which can be induced in a quiescent state by growth factors deprivation for 2 days (referred to as G_0_ cells). G_0_ cells were then treated or not with 4OHT for 2 hours to induce DSBs. The efficiency of DSB induction at each AsiSI site was measured in these two conditions by ChIP-sequencing of proliferating and G_0_-arrested cells using the anti-γH2AX antibody. Similarly to ChIP data already available for U2OS cells [22], γH2AX showed a typical pattern with signals encompassing the DSBs for 1-2Mb around the AsiSI sites, with the typical signal drop occurring exactly at the restricted AsiSI sites (Figure 3, and Supplementary Figure 2). Most importantly, we confirmed the results by analyzing 150 γH2AX peaks and found that γH2AX mapped with similar efficiency in both G_0_ and proliferating cells (Figure 3 and Supplementary Figure 2 and Table 3). From these observations we assessed that the efficiency of DSBs induced in either proliferating or G_0_-arrested cells is largely similar. Next, we followed γH2AX and 53BP1 foci formation by immunofluorescence in damaged G_0_-arrested cells. We found that, similarly to proliferating cells, G_0_-arrested cells exposed to 4OHT for 2 hours showed a drastic induction of γH2AX and 53BP1 foci. However, we found that DSB in G_0_-arrested cells were not repaired, with a persistent accumulation of γH2AX and 53BP1 foci up to 5 days after DSB induction, thus suggesting an impaired repair proficiency (Figure 4A). Accordingly, γH2AX ChIP data showed a sustained accumulation of γH2AX signal at the AsiSI sites (Figure 4B). We cannot exclude that DSBs might normally repaired in quiescent cells but fail to recover normal chromatin arrangement after repair. However, sustained expression of the P-p53-p21 axis was observed, suggesting that the DDR p53 pathway operates in G_0_-damaged cell. Interestingly, p21, which was present at high levels in quiescent cells, was further up-regulated after damage (Figure 4C). Moreover we found that damaged G_0_-arrested cells underwent to apoptosis after 3 days of OHT treatment, as documented by a robust increase of the cleaved PARP1 protein in damaged cells. PARP1 cleavage was not observed in vehicle treated undamaged cells (Veh) that could be kept in culture up to 10 days (Figure 4D and data not shown).

Next we investigated whether the lack of DNA repair efficiency was a consequence of different expression levels of DDR genes in G_0_-arrested cells compared to proliferating cells. We comparatively quantified expression levels of DDR genes in G_0_-arrested versus asynchronous proliferating cells by qRT-PCR and Western blot analysis. As shown in Figure 4E and 4F, G0-arrested cells expressed the analyzed DDR factors at comparable levels with proliferating cells except for RAD51 and ATR. The low expression levels of RAD51 and ATR are consistent with their role in G_1_/S phases. Data obtained showed that no significant differences were seen between G_1_/S and asynchronous proliferating cells.

From these findings we concluded that G_0_-arrested cells lack DNA repair proficiency, but retain the capability to activate DNA damage response.

### Cell-cycle reentry induces a delayed resolution of DSBs

**Figure 5:**
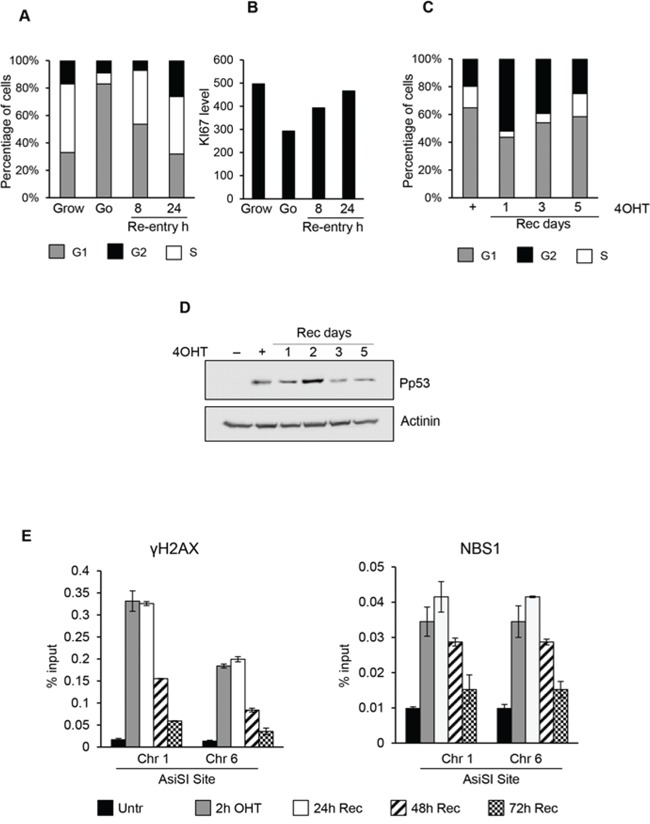
Cell-cycle re-entry induces a delayed resolution of DSBs In panel **A and B.** cell cycle profiles and Ki67 levels detected by flow cytofluorimetry of G_0_ MCF10-AsiSI-ER released into fresh medium and collected as indicated. **C.** Cell cycle distribution of G_0_-arrested MCF10A-AsiSI-ER cells treated for 2h with 4OHT, then released into fresh medium and collected as indicated. DNA content of propridium iodide stained cells was determined by flow cytofluorimetry. **D.** Western blotting of MCF10A-AsiSI-ER cells treated as above. **E.** ChIP against γH2AX and NBS1 in MCF10A-AsiSI-ER treated for 2h with 4OHT, then released into fresh medium, collected as indicated and analyzed by qPCR. Data are from independent experiments with SD (*n* = 3).

Our findings demonstrated that DSB occurring in G_0_-arrested cells are not repaired. We then sought to determine whether reentry of G_0_-damaged cells in cell cycle progression might recover DNA repair proficiency. First, we monitored the cell cycle re-entry of MCF10AsiER cells upon 2 days of starvation. Cells were grown in minimal medium for 2 days and then cell cycle re-entry was induced by addition of medium containing growth factors (hydrocortisone, EGF, insulin, cholera toxin). Flow cytometry analysis revealed an increase of S phase cells 8 hours after growth factors addition, with a concomitant increase of Ki67 levels compared to starved cells; moreover after 24 h, percentages of cell cycle phases and Ki67 levels were largely similar to growing control cells (Figure 5A and 5B).

Damaged G_0_-arrested cells showed an accumulation in G_2_ phase with a delayed cell-cycle re-entry (Figure 5C) with abnormal accumulation on both G_1_ and G_2_ phases. As shown in Figure 5D, P-p53 levels increased after DNA damage and accumulation of P-p53-Ser15 was detected up to 2 days after DSBs followed by a sharp decline at 3 and 5 days after recovery, suggesting resolution of induced damage. Finally we monitored accumulation of the DNA repair factors γH2AX and NBS1 at specific AsiSI sites at different time-points after cell-cycle reentry. ChIP data demonstrated that γH2AX and NBS1 factors were rapidly recruited to the AsiSI sites. However, compared to DNA damage in proliferating cells, in damaged G_0_-arrested cells we found a persistent accumulation of both γH2AX and NBS1 at DSBs (Figure 5E).

Collectively, these data clearly indicate that cell cycle re-entry of damaged G_0_-arrested cells induces delayed resolution of DSBs compared to proliferating damaged cells; following recruitment of repair factors, the progressive reduction in time of accumulation of these factors in G_0_-damaged cells was clearly delayed compared to what observed in asynchronously proliferating MCF10 cells.

### Efficient resolution of DSB induced at G_1_/S phase

**Figure 6:**
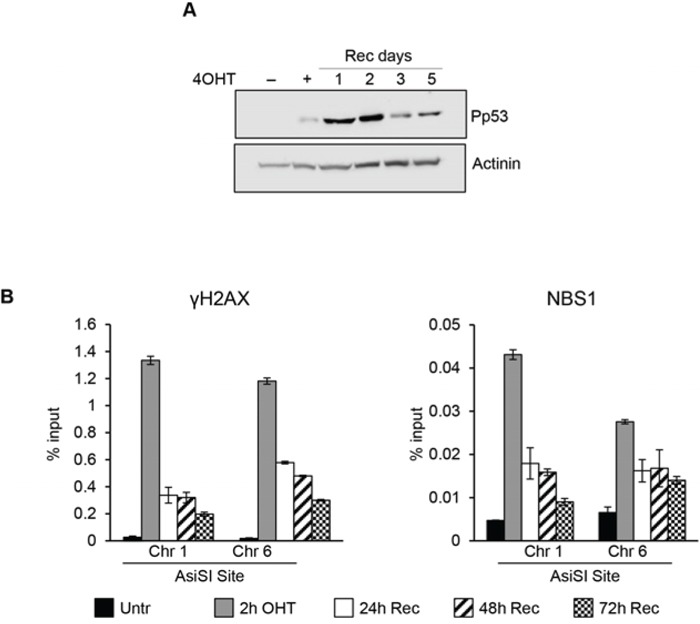
DSBs induced at G1/S phase Synchronized cells were exposed for 2 hr to 4OHT and then allowed to recovery for the indicted times. **A.** total cell extracts from G1/S phase MCF10A-AsiSI before and at the indicated times after 4OHT removal were probed with anti-phospho-p53 and actinin as loading control. Panel **B.** ChIP against γH2AX and NBS1 in MCF10-AsiSI-ER analyzed by qPCR. Data are from independent experiments with SD (*n* = 3).

Our findings demonstrated that cell cycle reentry of G_0_-damaged cells allows resolution of DSBs albeit with a delayed efficiency compared to proliferating cells, suggesting that transition to G_1_/S phases might be required for DSB resolution. We then sought to determine repair proficiency in synchronized G_1_/S-damaged cells. As shown by FACS data, 8 hours after cell cycle re-entry, G_0_-arrested MCF10-AsIER cells were synchronized in late G_1_/S phase (Figure 5A). Synchronized cells were exposed for 2 hours to 4OHT treatment and then allowed to recover: cell samples were collected at different times after the 4OHT pulse and analyzed for p53 activation and accumulation of DSB factors at specific AsiSI sites. G_1_-damaged cells exhibited a robust p53 activation (Figure 6A), and accumulation of the DNA repair factors (γH2AX and NBS1) at specific AsiSI sites at different time-points following DNA damage. ChIP data showed that γH2AX and NBS1 were rapidly recruited to the AsiSI sites and, most importantly, reduction of these DSBs factors followed a kinetics similar to that observed in asynchronously proliferating cells (compare Figure 6B and Figure 2C). The data demonstrated that, unlike damaged G_o_-arrested cells, G_1_-damaged cells exhibit efficient resolution of DSBs.

**Figure 7:**
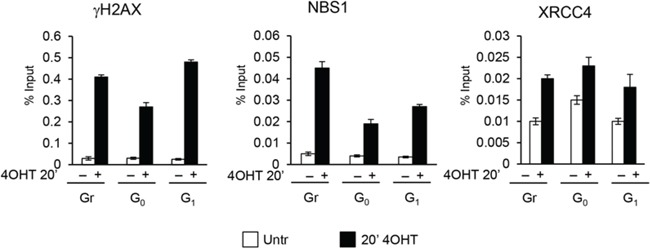
ChIP analysis with γH2AX, NBS1 and XRCC4 antibodies in MFC10AsiSI-ER cells after a short pulse of 4OHT treatment (20′) The values reported were calculated as percentage of input. Error bars indicate SD for three independent experiments.

Because DSBs assembly of DDR repair factors occurs within minutes following the DNA damage event, we comparatively determined the timing of γH2AX, NBS1 and XRCC4 recruitment in cells exposed to AsiSI-damage for a short period of time (20′) in asynchronously proliferating, G_0_-, or G_1_-arrested cells. Figure 7 shows that early recruitment of these factors is largely similar in all the three cell populations analyzed. Thus, the initial recognition of DSBs and assembly of DSB factors is largely similar regardless of the cell cycle phase during which the DSB is produced.

## DISCUSSION

Here we report a comparative study of DSB response occurring at specific stages of the cell cycle. We generated a human normal non-tumorigenic epithelial MCF10A cell line expressing the estrogen receptor-inducible AsiSI restriction enzyme, which allows the study of the wave of repair events occurring during specific stages of the cell cycle. We found that DSBs occurring in G_0_ cells are irreparable and G_1_/S transition is required for complete DNA damage resolution. We demonstrated that the G_0_ cells retain a functional DDR but, lacking DNA repair competence, they may accumulate DNA damage, which could reach critical levels and triggers the apoptotic cascade.

In agreement with previous studies in U2OS [22, 30–33], AsiSI dependent DSBs in proliferating MCF10A induce canonical DDR activation, which is followed by progressive resolution of DSBs. Conversely, we found that DSB induction in G_0_-arrested cells leads to efficient γH2AX and 53BP1 foci formation, but differently from asynchronous cells, damage is never repaired. Damaged G_0_-arrested cells show a robust and irreversible activation of the phospho-p53/p21 axis, and undergo apoptosis one week after DSBs induction. Through ChIP-sequencing we found that proliferating and G_0_ damaged cells showed the same number of DSB domains with similar enrichment of γH2AX, demonstrating that the efficiency of DSBs induced in either proliferating or G_0_-arrested cells is largely similar. Moreover, ChIP experiments revealed that in G_0_ damaged cells the levels of γH2AX, NBS1 and XRCC4 recruited to DSBs were largely similar to synchronous proliferating cells; thus, the cell cycle phase does not interfere with the initial steps of DNA damage response. However, the persistent accumulation of repair factors at DSBs indicates that DNA repair resolution is compromised in G_0_ cells. The inability of G_o_ cells to repair damage was not due to altered expression of DDR genes, since G_o_-arrested cells show similar expression levels of different repair factors when compared to proliferating cells, with the exception of RAD51 expression. Lack of RAD51 expression in G_o_ cells is consistent with the notion that HR, which relays on RAD51 activity is efficient in S and G_2_ cell cycle phase, but limited in G_0_/G_1_ [14–16].

Most notably, we find that cell cycle re-entry of G_0_ damaged cells restored DNA repair competence, but led to delayed resolution of DSBs compared to proliferating cells, suggesting that G_0_-damaged cells required G_1_/S transition to complete DSBs repair. Accordingly, resolution of DSBs induced in synchronized G_1_/S cells occurred with kinetic similar to that observed in proliferating cells.

Here we demonstrated that DDR activation does not depend on the phase of the cell cycle in which the DSB is generated. Similarly, a recent work reported that in IR-exposed fibroblasts, quiescence does not affect the DNA damage response, and activation of p53 and phosphorylation of γH2AX are similar between proliferating and quiescent cells [34].

Our data reveal that in G_0_ most of examined DDR factors are expressed at levels comparable to those observed in proliferating cells, and NHEJ is the main repair pathway since RAD51, a critical component of HR, is undetectable in Go. However, the G_1_/S transition is required to complete resolution of DSBs induced in G_0_ cells. A possible explanation is that some DSBs induced in G_1_ are repaired by HR as cells progress to S phase [35]. Clearly, different molecular mechanisms involved in DNA repair occurring at specific cell cycle phases have been evolved, and the DDR differs in mitotic and interphase cells. It has been shown that DDR is dampened during mitosis. During mitosis DDR is inhibited to prevent telomere fusion and entry into mitosis in the presence of unrepaired DNA can lead to cell death, thus DDR clearly differs in mitotic and interphase cells [25–28]

Mammalian tissues and organs of consist of different cell types, including dividing, non-dividing and stem cells. Terminally differentiated cells are permanently withdrawn from the cell cycle and partly resistant to apoptosis [36, 37]. d'Adda di Fagagna and collaborators showed that terminally differentiated astrocytes exhibit radio-resistance and strongly attenuated expression of most of DDR genes compared to undifferentiated progenitors [38]. It has been shown that in IR-exposed quiescent myoblast the ATM-p53 axis operates normally, while it is compromised in differentiated myotubes [39], indicating that the lack of a robust DDR and radio-resistance can be linked to the terminal differentiation and irreversible exit from the cell cycle. We cannot exclude, however, that differences in DNA damage response in cultured G_0_-arrested cells and in terminally differentiated non-proliferating cells are strictly cell type-specific and depend on the physiological context.

Our findings also help to dispel the dogma that completion of DNA damage repair is the essential condition for entry in the next phase of the cell cycle and stresses the notion the cell cycle position of a damage cell affects the repair competence. Further investigations are needed to understand mechanisms that coordinate repair in proliferating, quiescent and terminally differentiated cells to preserve genome integrity.

## MATERIALS AND METHODS

### Cell cultures and retroviral infection

MCF10A were cultured in 1:1 mixture DMEM-F12 supplemented with 5% horse serum, 10 μg/ml insulin, 0,5 μg/ml hydrocortisone, 100 ng/ml cholera enterotoxin, and 20 ng/ml epidermal growth factor, and incubated at 37°C in humidified atmosphere with 5% CO2. To generate MCF10Asi-ER cells the pBABE-HA-AsiSI-ER plasmid was transfected into 293T cells expressing the structural components for retrovirus packaging, medium was harvested after 36 h, filtered and used to infect MFC10 cells and selection performed using 1μg/ml puromycin; single cell clones were isolated and analyzed.

### Cell cycle synchronization

MCF10A-AsiSI cells were arrested in G_0_ by growth in minimal medium (1:1 mixture DMEM-F12 supplemented with 5% horse serum) for two days. To induce re-entry into cell cycle, the G_O_ arrested cells were cultured in complete medium and cell cycle re-entry was monitored by flow cytometry analysis and Ki67 content.

### Antibodies

The antibodies used for different applications in this study are listed in Supplementary Table S1.

### Flow cytometry analysis

To analyze the DNA profile, cells were fixed in methanol at −20°C and stained in hypotonic solution of 0,1% Na-Citrate, 50 μg/ml propidium iodide, 50 μg/ml RNAse and 0,00125% NP40 for 30′ at room temperature. For Ki67 quantification cells were permealyzed with 0,1% Triton X-100/PBS, blocked in 5% Bovine Serum Albumin/PBS and stained with the primary antibody anti-Ki67; then, cells were incubated with the secondary antibody Alexa647 Donkey anti-goat (Invitrogen) before propidium iodide staining. Cytofluorimetric acquisition and analysis were performed on a Becton Dickinson FACScalibur flow cytometer using FACSDiva, CellQuest Pro and ModFit LT 3.0 software.

### Western blot analysis

Whole-cell extracts were obtained using buffer F (10 mM TrisHCl pH 7.5, 150 mM NaCl, 30 mM Na4O7P2, 50 mM NaF, 5 mM ZnCl2, 0.1 mM Na3VO4, 1% Triton, 0.1mM PMSF). 50 μg of protein extracts were loaded and separated by SDS-PAGE and WB was performed with indicated antibodies.

### Immunofluorescence

Immunofluorescences of MFC10AsiSI-ER cells were performed as previously described. Briefly, cells were fixed in 4% paraformaldehyde in PBS, permeabilized in 0.1% Triton X-100 in PBS, pre-blocked in 2% BSA–3%NS-PBS for 30 min at room temperature, and then incubated for 1 h at 37° C with mouse anti-HA and rabbit anti-γH2AX for 30′ at 37°C anti-53BP1. Primary antibodies were detected by incubation with Cy3-coniugated anti-mouse or FITC-conjugated anti-rabbit antibody. Images were acquired using a Nikon Eclipse TE 2000-U microscope.

### Chromatin immunoprecipitation assays

ChIP experiments with chromatin extracts from MCF10-AsiSIER cells were performed as described [32]. IPs materials were analyzed in duplicate by quantitative PCR, using Syber Green 2X PCR Master Mix (Applied Biosystem). For qPCRs 3 μl out of 150μl immunoprecipitated DNA was used. The antibodies are listed in Table S1. After reversal of the crosslinks, the immunoprecipitated DNA was quantified by qPCR with the primer sets described in Table S2. For each ChIP assay a control amplicon from Chromosome X (Table S2) was used.

### Chip-sequencing, mapping and peak analysis

ChIP-seq libraries were prepared from 10 ng of ChIP (or Input) DNA with TruSeq ChIP Sample Prep Kit (Illumina) according to the manufacturer's instructions. Prior to sequencing, libraries were quantified using Quibit (Invitrogen) and quality-controlled using Agilent's Bioanalyzer. 50bp single-end sequencing was performed using Illumina HiSeq 2000 platform (Genomix4life S.R.L., Baronissi, Salerno, Italy) according to standard operating procedures. Alignments were performed with BWA [40] to hg18 using default parameters. SAMtools [41] and BEDtools [42] were used for filtering steps and file formats conversion. The peaks were identified from uniquely mapped reads without duplicates using MACS and the p-value cutoff used for peak detection was 1e-5. DNA Input was used as control. UCSC genome browser was used for data visualization. To plot data of average profiles around DSBs, AsiSI site positions were retrieved from the human genome (hg18). ChIP-seq counts were retrieved for 10 kb around each of these DSBs and averaged with a 200-bp window using a custom R-script [43]. ChIP-seq data were deposited to NCBI GEO and are available under accession number GSE71447

### RNA extraction and qRT-PCR quantification

RNA was extracted from MCF10A-AsiSIER cells using EuroGold Trifast (EuroClone). cDNA was generated using Quantitec Reverse Transcription Kit (Qiagen), according to manufacturer's protocol. Quantitative analysis was performed using SYBR Green 2X PCR Master Mix (Applied Biosystem). Each sample was run in triplicate and normalized to the expression of housekeeping beta-glucoronidase (GUS) gene as previously described [44]. Primers are presented in Supplementary Table S2.

## SUPPLEMENTARY FIGURES AND TABLES




