# Supplementary material for: Cell cycle-dependent resolution of DNA double-strand breaks
Source: Oncotarget. 2015 Dec 17;7(4):4949–60. doi: 10.18632/oncotarget.6644 (PMC4826256; doi:10.18632/oncotarget.6644)
Supplement: Supplementary file 1 [file oncotarget-07-4949-s001.pdf]

SUPPLEMENTARY FIGURES AND TABLES

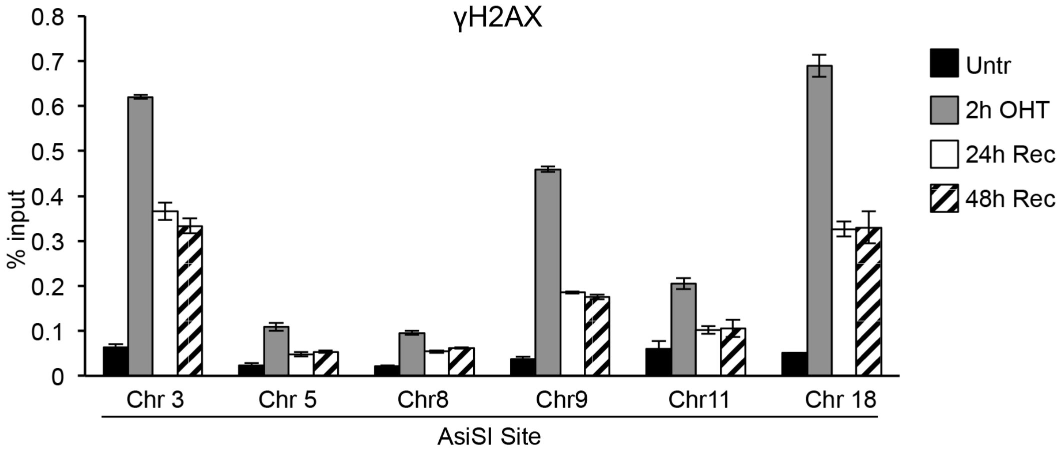

Supplementary Figure S1: Asynchronously growing MCF10A-AsiSI-ER were treated as described in Fig 2E and ChIP was carried out using γH2AX antibody and analyzed with primers located about 1 Kb away from AsiSI sites on the indicate chromosomes.

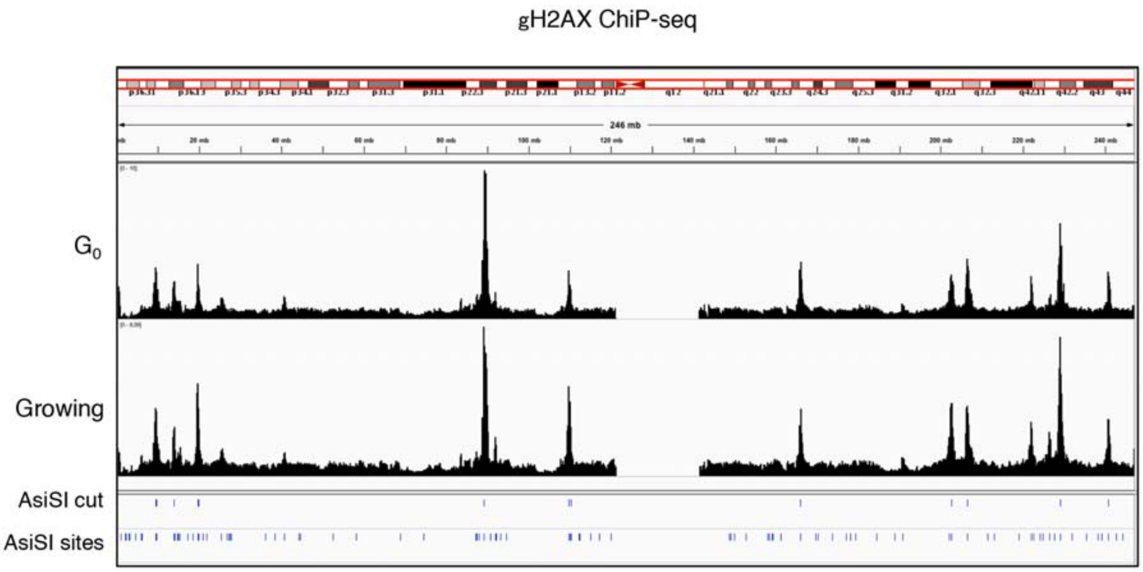

Supplementary Figure S2: ChIP-seq profiles of γH2AX at chromosome 1 in growing and G_0-arrested cells.

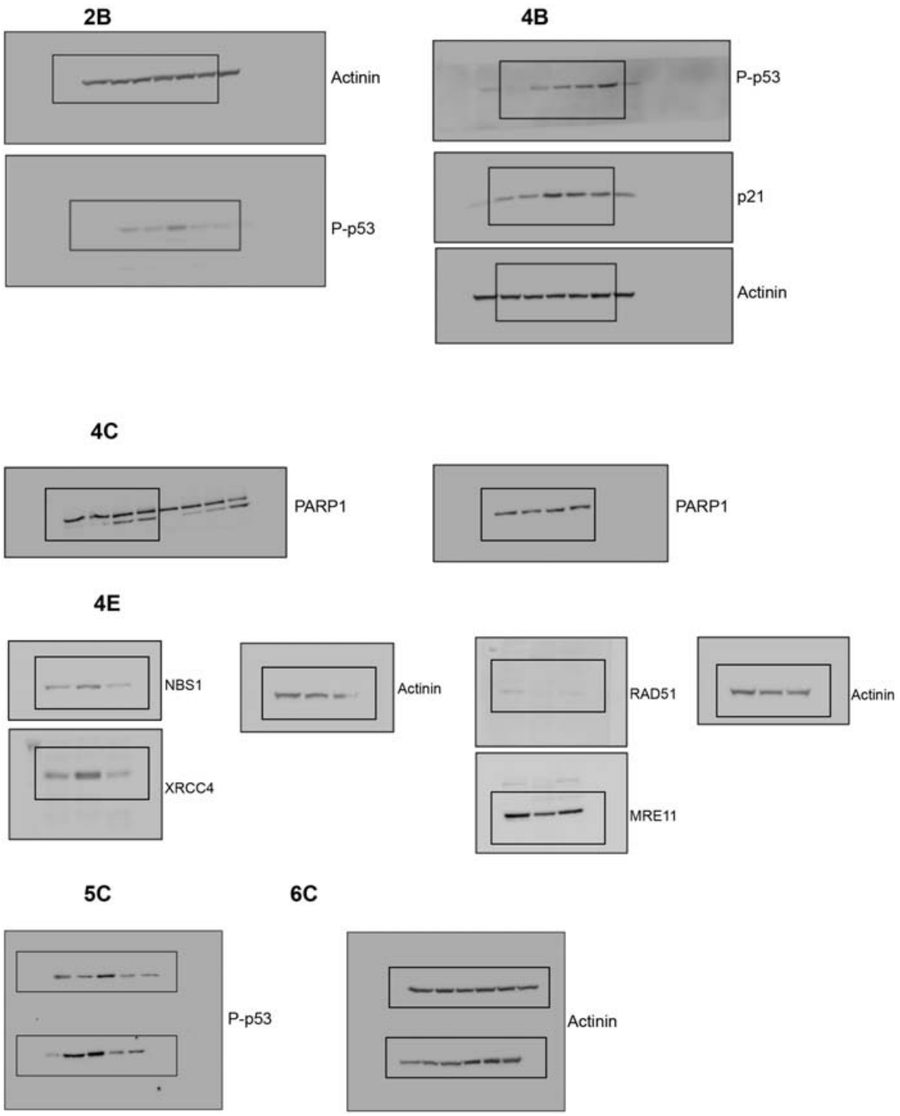

Supplementary Figure S3: Full scans of Western data.

Supplementary Table S1: Antibodies used in this work

Protein	Used for	Manufacturer
Actinin	WB	sc-17829, Santa Cruz
γH2AX (Ser139)	ChIP, IF	ab81299, Abcam
HA	IF	32-6700, Invitrogen
MRE11	WB	NB100-142, Novus Biological
p95 NBS1	ChIP, WB	ab32074, Abcam
P21 (CDKN1A)	WB	sc-397, Santa Cruz
P-p53 (Ser15)	WB	#9284, Cell Signaling
PARP-1	WB	sc-53643, Santa Cruz
RAD51	WB	sc-8349, Santa Cruz
XRCC4	ChIP, WB	ab145, Abcam
53BP1	IF	ab36823, Abcam
Ki67	Flow cytometry	sc-7846, Santa Cruz

Supplementary Table S2: Oligonucleotide primers used in this study

Figure 1, 2, 5 and 6

ChIP γH2AX

AsiSI site position (NCBI36/hg18)	Distance from AsiSI site	Forward	Reverse
Chr1: 89231183-89231191	1200bp	GACACCAAGGGCTGTTTTTC	TTTAAGACACATGTTTTCTCTACCTCA
Chr6: 69040490-690404914	1000bp	CTTGCCTCGCCTGTTAGC	GGGGAAATAGCCAGAACACA
ChrX: 87250472-87250540	No AsiSI site	TCATGGGTAGAAGTAACCTGG	CCTGGCCCATTTGTTCTTAC

Figure 1, 2, 5 and 6

ChIP NSB1

AsiSI site position (NCBI36/hg18)	Distance from AsiSI site	Forward	Reverse
Chr1: 89231183-89231191	250bp	GGGCTCGAATCGGATGTAT	GCAACCCAGTCCTCGTCCTA
Chr6: 69040490-690404914	180bp	CTGCTCTGTCAAACCTAAGAACC	CCCTTTTCAGCCCGGTAG
ChrX: 87250472-87250540	No AsiSI site	TCATGGGTAGAAGTAACCTGG	CCTGGCCCATTTGTTCTTAC

Figure 1

ChIP XRCC4

AsiSI site position (NCBI36/hg18)	Distance from AsiSI site	Forward	Reverse
Chr1: 89231183-89231191	1200bp	GACACCAAGGGCTGTTTTTC	TTTAAGACACATGTTTTCTCTACCTCA
Chr6: 69040490-690404914	1000bp	CTTGCCTCGCCTGTTAGC	GGGGAAATAGCCAGAACACA
ChrX: 87250472-87250540	No AsiSI site	TCATGGGTAGAAGTAACCTGG	CCTGGCCCATTTGTTCTTAC

Supplementary Figure 1

ChIP γH2AX

AsiSI site position (NCBI36/hg18)	Distance from AsiSI site	Forward	Reverse
Chr3: 52207202-52207209	1100bp	TCCCACTGCCACTCTGAAC	CAAGGCAGGTGAGGTGAGTC
Chr5: 68499664-68499725	1100bp	AACTCTCTTCCTGACCTAACTTCTGT	TCTGCATCAAAGGCCACA
Chr8: 116749804-116749812	1200bp	AAACCCACCAGTCCTTCTCA	CCAGGGAAGTCAGCAACTGT
Chr11: 100064603-100064716	1200bp	GCACAATTACATGATATGGCTTG	ACAGGCTCCTTTGAAGATGG

(*continued*)

AsiSI site position (NCBI36/hg18)	Distance from AsiSI site	Forward	Reverse
Chr9: 35900388-35900396	1400bp	AACCGCAGAATCAGATGGTC	CTCCCTCCACTCCTAGCTCA
Chr18: 7556711-7556719	1000bp	AATTAAAGATAAGGTTCCATAAGCACA	TTGTTAATATGAGCTTACATTGTCCTG

Figure 4
Quantitative RT-PCR

Gene	Forward	Reverse
MRE11	AAGATGATGAAGTCCGTGAGG	GAAGCAGACTCCTCTGACTGAGAT
RAD51	TGAGGGTACCTTTAGGCCAGA	CATCACTGCCAGAGAGACCA
53BP1	TGGTTCCATCAGTCAGGTCA	CCACTGACATTCCCAGAACA
ATM	TTTCTTACAGTAATTGGAGCATTTT	GGCAATTTACTAGGGCCATTC
DNA-PK	ACACGTTCATTGGCGAGAG	TGAAGACTGGGCTTCAGTACC
XRCC4	CTTGGGACAGAACCTAAAATGG	GACGTCTCAGGTAGTGAAGAATCA
KU70	GCCCTCCCTGTTCGTGTA	CTTGATGAGCAGAGCACTGAA
NBS1	TTATATTTTTGAATGCCAAACAGC	TCATTCTCTTCTGTTATCAACCTAGC
ATR	GACATTGGGCCTATATTGCAG	TGGTTTCTGAAGAGAAGCAAGA

Supplementary Table S3: AsiSI sites in MCF10A

See Supplementary Table S3
